# *Helicobacter pylori* Infection in Children with Phenylketonuria Does Not Depend on Metabolic Control and Is Not More Frequent Than in Healthy Subjects—A Cross-Sectional Study

**DOI:** 10.3390/children8080713

**Published:** 2021-08-19

**Authors:** Marek Walkowiak, Łukasz Kałużny, Renata Mozrzymas, Małgorzata Jamka, Bożena Mikołuć, Joanna Jagłowska, Ewa Starostecka, Roza Nurgaliyeva, Jarosław Walkowiak, Aleksandra Lisowska

**Affiliations:** 1 Department of Reproduction, Poznan University of Medical Sciences, Polna Str. 33, 60-535 Poznan, Poland; walkowiak.gpsk@gmail.com; 2 Department of Pediatric Gastroenterology and Metabolic Diseases, Poznan University of Medical Sciences, Szpitalna Str. 27/33, 60-572 Poznan, Poland; lkaluzny@ump.edu.pl (Ł.K.); mjamka@ump.edu.pl (M.J.); jarwalk@ump.edu.pl (J.W.); 3 Research and Development Center, Regional Specialist Hospital, Kamieńskiego Str. 73a, 51-124 Wrocław, Poland; renata.mozrzymas@gmail.com; 4 Department of Pediatrics, Rheumatology, Immunology and Metabolic Bone Diseases, Medical University of Bialystok, Waszyngtona Str. 17, 15-274 Bialystok, Poland; bozenam@mp.pl; 5 Department of Pediatrics, Hematology and Oncology, Medical University of Gdansk, Dębinki Str. 7, 80-211 Gdansk, Poland; jjaglowska@uck.gda.pl; 6 The Regional Center of Rare Diseases, Polish Mother’s Memorial Hospital Research Institute, Rzgowska Str. 281/289, 93-338 Łodź, Poland; ewastarostecka@wp.pl; 7 Department of Normal Physiology, West Kazakhstan Marat Ospanov Medical University, Maresyev Str. 68, Aktobe 030019, Kazakhstan; nuroz61@mail.ru; 8 Department of Clinical Auxology and Pediatric Nursing, Poznan University of Medical Sciences, Szpitalna Str. 27/33, 60-572 Poznan, Poland

**Keywords:** inborn errors of metabolism, phenylalanine, paediatrics, anthropometry

## Abstract

In a small preliminary study, phenylketonuria and poor metabolic control were suggested as risk factors for *Helicobacter pylori* infection in children as detected with an antigen stool test. We aimed to determine *Helicobacter pylori* prevalence in an adequately sized group of individuals with phenylketonuria and healthy subjects using the standard gold test (urea breath test). Further, we correlated *Helicobacter pylori* infection with metabolic control. The study comprised 103 individuals with phenylketonuria and 103 healthy subjects on whom a ^13^C urea breath test was performed. Blood phenylalanine levels in the preceding year were analysed. The infection rate did not differ between individuals with phenylketonuria and healthy subjects (10.7% vs 15.5%; *p* = 0.41). The frequency of testing and phenylalanine concentrations of *Helicobacter pylori*-positive and *Helicobacter pylori*-negative patients with phenylketonuria did not differ (*p* = 0.92 and *p* = 0.54, respectively). No associations were detected for body mass index or metabolic control. Forward stepwise regression models revealed that age (*p =* 0.0009–0.0016) was the only independent correlate of *Helicobacter pylori* infection with a relatively low fraction of the variability of the condition being explained (adjR^2^ = 0.0721–0.0754; model *p* = 0.020–0.023). In conclusion, *Helicobacter pylori* infection in phenylketonuria is not more frequent than in the general population. Moreover, it does not depend on metabolic control.

## 1. Introduction

Phenylketonuria (PKU) is an inborn error of metabolism that results in the abnormal metabolism of phenylalanine (Phe). Untreated or not adequately treated PKU can result in behavioural problems, mental disorders, intellectual disability and seizures. In general, the overall prognosis for patients with PKU is good [1,2]. Adequately treated subjects may have no detectable neurological or developmental problems. Without any doubt, low Phe levels are associated with good neurological performance [3]. However, recent reports suggest that the risk of comorbidities is higher than in the general population [3,4,5,6]. This seems justified since the compliance decreases with age [7,8,9].

Burton et al. [4] retrospectively analysed records of 3691 PKU patients and 18,455 matched non-PKU subjects in a case-controlled adult study (≥20 years old) based upon United States insurance claims databases between 1998 and 2014 to assess the prevalence of comorbidities. The adjusted prevalence ratios (PRs) of 15 conditions studied (alopecia, anaemia, asthma, eczema, oesophageal disorders, gallbladder disease, gastritis/esophagitis, kidney calculus, osteoporosis, overweight, renal insufficiency, rhinitis and urticaria) were significantly higher for the PKU cohort (*p* ≤ 0.001 for all). The highest adjusted PR (*p* < 0.001 for both) were for renal insufficiency with hypertension (PR (95% confidence intervals, 95% CI) = 2.20 (1.60–3.00)) and overweight (PR (95% CI) = 2.06 (1.85–2.30)). Trefz et al. [5] conducted a similar analysis in Germany, which comprised 377 adult PKU patients and 3770 matched controls. The highest PRs (1.6–2.3) were documented for major depressive disorders, chronic ischemic heart disease, asthma, dizziness and giddiness, diabetes mellitus, infectious gastroenteritis and colitis, and reactions to severe stress and adjustment disorders.

Oztürk et al. [10] suggested a higher risk of *Helicobacter pylori* (HP) infection in children with PKU than that in healthy peers (28.1% vs 9.4%). However, the control group of healthy subjects was very small. A dramatically higher frequency rate was documented in patients with mean Phe levels above 600 µmol/L for the preceding two years (51.8%) than those with lower concentrations (10.2%). Therefore, we aimed to determine the prevalence of HP in an adequately sized group of patients with PKU and thoroughly selected healthy peers. In addition, we correlated the presence of infection with individually defined metabolic control.

## 2. Materials and Methods

The study comprised patients with PKU treated at five centres in Poland. The inclusion criteria included a diagnosis of classical PKU, continuous treatment from birth and willingness to participate in the study. The exclusion criteria were chronic diseases, which could influence Phe levels. All subjects with PKU were continuously receiving Phe-free formula. None of them were on additional treatment (e.g., BH4, PEG-PAL formulation). None of the patients had a detectable neurological impairment or intellectual problems. All patients were attending normal schools, study or work. The assessment of metabolic control was based on Phe levels obtained within 12 months preceding the study. Phe concentrations were expressed as milligrams per decilitre (mg/dL). The number of tests, median Phe level and the percentage of abnormal Phe concentrations (>6 mg/dL up to 12 years of age and >10 mg/dL for older patients) were calculated for each patient according to recent recommendations [11]. Similarly, the percentage of Phe concentration exceeding the range of 6, 10 and 12 mg/dL was considered. The control group (healthy subjects—HS) consisted of healthy peer relatives of patients from metabolic centres. None of the patients with PKU or HS had received antibiotics or gastric acid suppressors (e.g., proton pump inhibitors) for 3 and 12 months, respectively, before the urea breath test (UBT).

In all patients and healthy controls, body weight and height were measured, and body mass index (BMI) was calculated. BMI was recalculated for patients younger than 18 years according to International Obesity Task Force values (BMI-IOTF corrected) [12]. The presence of HP was assessed using the ^13^C isotope-labelled UBT. The ^13^CO_2_/^12^CO_2_ ratio measurement was carried out using a ^13^C-infrared isotope analyser system (IRIS, Wagner Analysen Technik, Bremen, Germany) as described earlier [13,14].

The sample size was calculated with the assumption of a 15% difference (half of the previously published [10]) in the prevalence of HP infection between the studied group (25% vs 10%), assuming type II error of 20% and type I error of 5%. The minimal numbers of patients and controls were defined as 100 for each group. The minimum sample size was calculated using G*Power software (University of Kiel, Kiel, Germany).

The mean and standard deviation (SD), median, and interquartile range (IQR) were calculated for all data. The Mann–Whitney U test was used to compare continuous variables between the groups. The Fisher’s test was used in the case of Boolean variables. The odds ratio (OR) was calculated to compare the frequency of HP infection in patients with PKU depending on age, BMI and metabolic control in the preceding year (according to (a) the median Phe concentration or (b) the percentage of abnormal Phe concentrations or the percentage of Phe concentrations higher, respectively, than (c) 6 mg/dL, (d) 10 mg/dL, (e) 12 mg/dL). Forward stepwise regression models were built to identify variables with the strongest relationship with HP infection. The models included: age, gender, BMI (IOTF adjusted) and metabolic control (as described for OR). Statistical significance was set at 0.05, and all tests were two-sided. Statistical analyses were carried out using Statistica 13.3 (TIBCO Software, Palo Alto, CA, USA).

The study was approved by the Bioethical Committee at Poznan University of Medical Sciences (protocol code: 1069/15 and date of approval: 3 December 2015); all the patients provided informed consent for their participation in this research project.

## 3. Results

The characteristics of the studied subjects are summarised in Table 1. Patients with PKU and HS did not differ in terms of basic parameters (age, gender, BMI and BMI-IOTF corrected). The mean and median Phe concentrations in subjects with PKU in the preceding year were 8.62 and 7.67 mg/dL. The mean and median ratio of abnormal Phe levels counted for 46.4% and 36.4%, respectively. There was no difference in the infection rate between patients with PKU and HS (10.7% vs 15.5%; *p* = 0.41).

Subjects with PKU infected with HP were older and had higher BMI values (*p* = 0.026 and *p* = 0.048, respectively). However, BMI values corrected for age (BMI-IOTF corrected values) did not differ (*p* = 0.73). The frequency of testing and metabolic control of HP-positive and HP-negative patients in the preceding year were very similar (Table 2).

The odds of being infected with HP were higher for adults than for children and adolescents (*p* = 0.032). However, no differences were detected for nutritional status (BMI) or metabolic control (median Phe concentration and ratio of abnormal results) in the preceding year (Table 3).

Forward stepwise regression revealed that, exclusively, age was the independent correlate of HP infection. The five models explained a relatively low fraction of the variability of condition (R^2^ = 0.1085−0.1117, adjR^2^ = 0.0721−0.0754, *p* for the model = 0.020−0.023, *p* for the determinant factor = 0.0009−0.0016).

## 4. Discussion

This is the first comparative study on the prevalence of HP infection in patients with PKU and healthy controls using a urea stable isotope breath test [13]. We did not confirm the higher HP prevalence in patients with PKU as suggested by Oztürk et al. [10]. However, they performed a faecal test for the detection of the HP antigen, whereas we applied UBT as a gold standard [13]. Moreover, the number of healthy controls in the above-mentioned study was very small (*n* = 32) [10]. In the present study, we defined an adequate sample size, and assessed a large cohort of patients with PKU (*n* = 103) and matched healthy peers (*n* = 103).

The infection rate in patients with PKU and HS in the present study was not different. Similarly, the prevalence of HP did not differ between selected age subgroups from our study and recent country-based publications comprising healthy peers (Table 4). Żabka et al. [15] studied 356 healthy children aged 3–18 years, whereas Szaflarska-Popławska and Soroczyńska-Wrzyszcz [16] evaluated 3067 healthy teenagers (aged 13–17 years). In both studies, carried out in the general population, the prevalence of HP diagnosed by UBT was not significantly higher than that found in children and teenagers with PKU in our study [15,16]. Similarly, Tacikowski et al. [17], in a group of 148 adults, performed UBT and did not find a higher rate of infection than we noted in our adult patients. However, the authors assessed patients with dyspepsia with a mean age of 45 years (on average 10 years older than our control adult subjects). Having in mind the limitations mentioned above, the thoroughly selected control group in the present study seems to be of high clinical significance. However, the prevalence of infection in other Polish studies is similar and confirms the obtained results [18,19]. Interestingly, the prevalence of HP infection in the control group of Oztürk et al. [10] was significantly different (9.4%) than that in two large groups of children (*n* = 885 and *n* = 101) studied in Turkey (63.2% and 47.2%, respectively), based upon a histopathological assessment [20,21]. Although the prevalence of gastritis/esophagitis was suggested to be higher in American adult patients with PKU [4], gastritis and duodenitis were not documented to be more frequent in German patients [5] as compared to the prevalence of gastritis/esophagitis in healthy controls. However, patients with chronic diseases undergo more thorough controls, and are more likely to be diagnosed with other diseases. Therefore, one should consider the (relative) overestimation of morbidity in PKU as compared to healthy subjects in analyses based upon health records.

In subjects with PKU, we did not find any association between metabolic control and the prevalence of HP infection. Our results significantly contradict the findings by Oztürk et al. [10], who found five times more frequent HP infection in individuals with PKU with worse metabolic control than in those with better metabolic control (subjects with a median concentration higher or lower than 600 mmol/L, respectively) in their study. PKU diet compliance definitely influences neurological impairment and may impact nutritional status [22]. However, it is difficult to find a link to a potentially higher prevalence of HP in patients with PKU that are not significantly intellectually impaired. However, it should be considered that 15% of the PKU patients in the study by Oztürk et al. [10] were aged 2–8 years at diagnosis, which was made due to them being developmentally delayed. Besides, in our study, the mean age of individuals with PKU and HS was 15.97 ± 10.22 years and 15.92 ± 10.19 years, respectively, while Oztürk et al. [10] recruited younger children (PKU group: 8.2 ± 6.7 year, outpatient controls: 9.6 ± 4.7 years, respectively). Similarly to a previous PKU study [10], none of the patients were symptomatic.

The strengths of our study are that it includes the largest studied population of patients with PKU and controls, the use of a non-invasive gold standard technique and well-defined metabolic control. The limitations comprise the lack of analysis of family history, socioeconomic status, potential risky behaviours (such as consuming raw vegetables, drinking unboiled water, using collective catering facilities) [16] and the current intelligence scale. However, none of our patients were handicapped. Their previous IQ results were within the normal range for the population.

## 5. Conclusions

In conclusion, the HP infection rate in treated PKU is not higher than in the general population. Moreover, it does not depend on recent metabolic control.

## Figures and Tables

**Table 1:** Basic characteristics of patients with phenylketonuria (PKU) and healthy subjects (HS).

Parameter	PKU (*n* = 103)	HS (*n* = 103)	*p*
	*N* (%)		
Gender			1.00
Female	58 (56.3)	58 (56.3)	
Male	45 (43.7)	45 (43.7)	
	Mean ± SD Median (1st–3rd quartile)		
Age (years)	15.97 ± 10.22 12.86 (8.67–18.94)	15.92 ± 10.19 12.80 (8.55–19.12)	0.99
BMI (kg/m^2^)	20.58 ± 5.00 19.82 (16.29–23.12)	19.77 ± 3.98 19.50 (16.06–22.62)	0.44
BMI-IOTF corrected (kg/m^2^)	23.00 ± 4.10 22.07 (20.13–24.37)	22.19 ± 2.93 21.82 (19.82–23.68)	0.28
Number of Phe tests/year	10.3 ± 7.7 8 (5–14)	-	-
Phe concentration (mg/dL) in the preceding year	8.62 ± 4.85 7.67 (4.68–11.40)	-	-
* Abnormal Phe concentrations (%)	46.4 ± 38.0 36.4 (10.6–84.0)	-	-
Phe concentrations > 6 mg/dL (%)	62.8 ± 34.7 72.7 (30.4–100)	-	-
Phe concentrations > 10 mg/dL (%)	35.4 ± 36.9 23.1 (0–69.0)	-	-
Phe concentrations > 12 mg/dL (%)	23.3 ± 32.3 6.7 (0–39.3)	-	-

BMI—body mass index; IOTF—International Obesity Task Force; *n*—number; Phe—phenylalanine; SD—standard deviation; * 6 mg/dL up to 12 years of age and 10 mg/dl for older patients.

**Table 2:** Clinical characteristics of patients with phenylketonuria infected (HP-positive) and uninfected (HP-negative) with *Helicobacter pylori*.

Parameter	HP-Positive (*n* = 11)	HP-Negative (*n* = 92)	*p*
	*N* (%)		
Gender			1.00
Female N (%)	6 (54.5)	52 (56.5)	
Male N (%)	5 (45.5)	40 (43.5)	
	Mean ± SD Median (1st–3rd quartile)		
Age (years)	24.84 ± 11.54 25.79 (16.54–31.71)	14.90 ± 9.58 12.26 (8.43–17.26)	0.0026
BMI (kg/m^2^)	23.41 ± 5.50 22.84 (19.64–26.15)	20.24 ± 4.85 19.62 (16.18–22.67)	0.048
BMI (kg/m^2^) (IOTF corrected)	23.67 ± 5.37 22.84 (20.30–26.49)	22.92 ± 3.96 22.05 (20.16–24.10)	0.73
Number of Phe tests/year	9.2 ± 5.2 7 (5–13)	10.4 ± 7.9 8 (5–13)	0.92
Phe concentration (mg/dL) in the preceding year	9.26 ± 4.78 8.29 (6.63–10.17)	8.54 ± 4.88 7.65 (4.64–11.67)	0.54
* Abnormal Phe concentrations (%)	37.0 ± 30.0 28.6 (20.0–52.4)	47.5 ± 38.9 46.4 (9.1–85.7)	0.56
Phe concentrations > 6 mg/dL (%)	69.6 ± 32.1 77.8 (55.0–100)	61.9 ± 35.0 72.1 (29.8–100)	0.54
Phe concentrations > 10 mg/dL (%)	36.4 ± 30.5 28.6 (20.0–52.4)	35.3 ± 37.8 21.5 (0–69.2)	0.56
Phe concentrations > 12 mg/dL (%)	21.2 ± 28.6 0 (0–35.6)	23.6 ± 32.9 6.7 (0–38.8)	0.77

BMI—body mass index; IOTF—International Obesity Task Force; *N*—number; Phe—phenylalanine; SD—standard deviation; * 6 mg/dL up to 12 years of age and 10 mg/dl for older patients.

**Table 3:** The odds of being infected by *Helicobacter pylori* depending on age, body mass index (BMI) and metabolic control in the preceding year in patients with phenylketonuria.

Patients		OR (95% CI)	*p*
Older vs younger than 18 years		4.0571 (1.1249–14.6333)	0.032
With BMI higher vs lower than 25 kg/m^2^		1.35 (0.3275–5.5650)	0.68
Median Phe concentration higher vs lower than respective cut-off level for age		0.7108 (0.1946–2.5963)	0.60
Percentage of abnormal results higher than	40%	0.3590 (0.0896–1.4393)	0.15
	50%	0.3750 (0.0935–1.5032)	0.17
	70%	0.6107 (0.1518–2.4570)	0.49

BMI—body mass index; OR—odds ratio; Phe—phenylalanine; 95% CI—95% confidence intervals.

**Table 4:** Prevalence of *Helicobacter pylori* (HP) infection in patients with phenylketonuria (PKU) and in healthy subjects (HS) in Poland.

Age Group	HP Infection Rate (%)			*p*
	Present Study		Other Studies	
	PKU	HS	HS	
3–6 years	0	0		1.00
			3.7 [15]	1.00
7–12 years	2.7	5.4		0.62
			11.0 [15]	0.13
13–18 years	13.3	16.7		1.00
			15.0 [15]	1.00
			23.6 [16]	0.28
Young and middle-aged adults	22.2	29.6		0.76
			35.8 [17]	0.19

## Data Availability

The data presented in this study are available on request from the corresponding author. The data are not publicly available due to the disagreement of the study participants.
